# Modifiable and non-modifiable risk factors for obstetric anal sphincter injury in a Norwegian Region: a case–control study

**DOI:** 10.1186/s12884-022-04621-2

**Published:** 2022-04-01

**Authors:** Ragnhild Klokk, Kjersti S. Bakken, Trond Markestad, Mads N. Holten-Andersen

**Affiliations:** 1 Department of Pediatrics, Innlandet Hospital Trust, Anders Sandvigsgate, 2619 Lillehammer, Norway; 2 Women’s Clinic, Innlandet Hospital Trust, Lillehammer, Norway; 3 Centre for International Health, University of Bergen, Bergen, Norway; 4 Department of Research, Innlandet Hospital Trust, Brumunddal, Norway; 5 Department of Clinical Medicine, Faculty of Medicine, University of Oslo, Oslo, Norway

**Keywords:** Amniotomy, Birth, Birth injury, Modifiable risk factor, OASI, Obstetric anal sphincter injury, Vaginal delivery

## Abstract

**Background:**

Obstetric anal sphincter injury (OASI) is a common and severe complication of vaginal delivery and may have short- and long-term consequences, including anal incontinence, sexual dysfunction and reduced quality of life. The rate of OASI varies substantially between studies and national birth statistics, and a recent meta-analysis concluded that there is a need to identify unrecognized risk factors. Our aim was therefore to explore both potential modifiable and non-modifiable risk factors for OASI.

**Methods:**

We performed a case–control study in a single center maternity clinic in South-Eastern Norway. Data were extracted retrospectively from an institutional birth registry. The main outcome measure was the occurrence of the woman’s first-time 3^rd^ or 4^th^ degree perineal lesion (OASI) following singleton vaginal birth after 30 weeks’ gestation. For each woman with OASI the first subsequent vaginal singleton delivery matched for parity was elected as control. The study population included 421 women with OASI and 421 matched controls who gave birth during 1990–2002. Potential risk factors for OASI were assessed by conditional logistic regression analyses.

**Results:**

The mean incidence of OASI was 3.4% of vaginal deliveries, but it increased from 1.9% to 5.8% during the study period. In the final multivariate regression model, higher maternal age and birthweight for primiparous women, and higher birthweight for the multiparous women, were the only non-modifiable variables associated with OASI. Amniotomy was the strongest modifiable risk factor for OASI in both primi- (odds ratio [OR] 4.84; 95% confidence interval [CI] 2.60–9.02) and multiparous (OR 3.76; 95% CI 1.45–9.76) women, followed by augmentation with oxytocin (primiparous: OR 1.63; 95% CI 1.08–2.46, multiparous: OR 3.70; 95% CI 1.79–7.67). Vacuum extraction and forceps delivery were only significant risk factors in primiparous women (vacuum: OR 1.91; 95% CI 1.03–3.57, forceps: OR 2.37; 95% CI 1.14–4.92), and episiotomy in multiparous women (OR 2.64; 95% CI 1.36–5.14).

**Conclusions:**

Amniotomy may be an unrecognized independent modifiable risk factor for OASI and should be further investigated for its potential role in preventive strategies.

## Introduction

Most women experience perineal trauma of varying severity when giving vaginal birth [1]. Severe perineal lesions, referred to as obstetric anal sphincter injury (OASI), are diagnosed in as many as 11% of vaginal deliveries, but with significant variation between studies and national birth statistics [1–5]. The true incidence rate may be as high as 26% because the injuries can be overlooked at the delivery wards or be occult [4, 6]. Apart from the immediate perineal pain, OASI often has short- and long-term consequences including negative impact on sexual life and quality of life in general, including anal incontinence [7–10].

Adequate clinical examination following delivery is pivotal in the diagnosis of OASI [11, 12], and increased awareness and training of health care personnel have resulted in a doubling of detection rates [2, 12]. Alongside the focus on detection, prevention has gained increasing attention. Obstetric training programs for midwives with emphasis on potential preventive measures, such as attention to birth position and perineal massage during the second stage of labor, have been suggested as ways of decreasing the risk of OASI [13–15]. Implementation of a preventive program in five maternity clinics in Norway resulted in a decreased prevalence of OASI [16], as has similar programs in more recent studies in other European countries [17–20]. However, the evidence of persistent efficacy of preventive programs is weak, partly because the existing studies were assessed shortly after their introduction [21]. In a study involving the four large Nordic countries over seven years, a lasting reduction was only observed in Norway [22].

Established risk factors for OASI include primiparity, vaginal birth after caesarean delivery, advanced maternal age, high birthweight, fetal occiput posterior presentation, induction and augmentation of labor, instrumental delivery, increased duration of second stage of labor, episiotomy, and Asian ethnicity [1, 2, 23, 24]. A meta-analysis published in 2020 showed that the incidence of OASI remains high, and the need to search for hitherto unrecognized and potentially modifiable risk factors was highlighted [1]. We aimed at exploring both modifiable and non-modifiable risk factors in a large retrospective case–control study based on a regional cohort where detailed information related to maternal, pregnancy, delivery, and fetal characteristics had been collected prospectively.

## Methods

### Study design, setting and participants

At Innlandet Hospital Trust, Lillehammer, Norway, detailed information on maternal health, pregnancy, delivery, and the postpartum period until discharge is prospectively registered in a perinatal database. This hospital covers virtually all births in a region with a population of around 90,000 people at the time of the study; around 23,000 lived in the city Lillehammer and the others in rural areas with small towns. The women were registered in the perinatal database at 18–20 weeks’ gestation when they met for the routine ultrasound assessment. This study included all deliveries that occurred from January 1^st^,1990 through December 31^st^, 2002. From the database we identified singleton vaginal deliveries with gestational age (GA) > 30 weeks where women for the first time were diagnosed with perineal rupture. The data were quality assured and expanded by scrutinizing delivery protocols, charts, and patient records. Women with 3^rd^ and 4^th^ degree OASI were defined as cases, and we selected the next vaginal singleton delivery with the same parity and GA > 30 weeks without OASI as a matched control. Writing of the manuscript was done according to the STROBE checklist for the reporting of cohort, case–control and cross-sectional studies. The research project was approved by the Norwegian Social Science Data Services (project number: 2614) and The Norwegian Data Protection Authority (reference code: 95/2691–2 GSØ). The study was financed through Innlandet Hospital Trust research fund, grant number 150434.

### Definitions and interventions

OASI was diagnosed according to the International Classification of Diseases (ICD) 9 definition 664.2 and 664.3 (similar to ICD 10 codes O70.2 and O70.3). The included cases were diagnosed at the time of the tear by the midwife or physician in charge of the delivery and subsequently confirmed by a specialist in obstetrics and gynecology. Consequently, women with potential delayed diagnosis of OASI are not included.

In addition to degree of perineal rupture, modifiable and non-modifiable variables regarding the infant, mother and birth process were registered. Non-modifiable variables included birth weight (gram), length (cm), head circumference (cm), gestational age (GA) (weeks), and maternal age (years), parity, duration of the first and second stage of labor (minutes), and fetal presentation (occiput posterior, occiput anterior, deep transverse, breech). Modifiable variables included the mother’s birth position (supine/sitting, side bearing, standing, kneeling, or on stool), induction of labor (yes/no), amniotomy (yes/no), episiotomy (mediolateral, yes/no), augmentation with oxytocin (yes/no), and instrumental delivery by vacuum extraction (yes/no) or forceps (yes/no).

Methods used for induction of labor were based on the Bishop scores and included membrane sweeping, transcervical Foley catheter, prostaglandin vaginal tablets, amniotomy or/and augmentation with oxytocin. Amniotomy was performed in births with a spontaneous onset when continuous surveillance of the fetus with a scalp-electrode or an examination of the amniotic fluid was considered necessary. Furthermore, amniotomy was employed before augmentation with oxytocin in cases of labor dystocia. Indications for performing an episiotomy included imminent fetal asphyxia, preterm birth. Instrumental vaginal delivery included vacuum extraction and the use of forceps at the physician’s discretion.

Birthweight was categorized into quartiles: < 3300, 3300–3659, 3660–4039, and ≥ 4040 g. Crown-heel length and head circumference were measured according to protocol. GA was estimated according to routine ultrasonography at 18–20 weeks of gestation at Lillehammer Hospital. Maternal age was categorized into the following three groups: < 25, 25–29, and ≥ 30 years. The cases and controls were stratified to primiparous (first birth) or multiparous (≥ second birth).

### Statistical analyses

Missing data were treated by listwise deletion. Continuous variables were tested for distributions of normality and described by means and standard deviations. Categorical variables were described by frequencies and proportions. We performed separate analyses for the primi- and multiparous pregnancies. In addition, we performed subgroup regression analyses for cases and controls giving their 3^rd^ birth or more. For the variables within each group, we analyzed differences between cases and controls with t-tests, Kruskal–Wallis or Chi^2^-tests. Significance level was set at 5%. Correlation matrices demonstrated covariation between weight, length, and head circumference of the infant, and only birthweight was used in logistic regression analyses. We used univariate conditional logistic regression analyses when assessing associations between exposure variables and OASI. Apart from birth length and head circumference, all registered modifiable and non-modifiable variables were tested in the univariate regression analyses. Subsequently, we built risk-factor models for OASI by using multivariate conditional logistic regression analyses progressing with a stepwise procedure. In this analysis we included variables that were significantly different between the cases and controls in the univariate analyses. We assessed multicollinearity by using variance inflation factor (VIF). We assessed interactions between amniotomy and the following variables: augmentation with oxytocin, episiotomy, and instrumental delivery by vacuum or forceps. STATA 16.1 software (STATA, College Station, TX, United States: StataCorp, 2020) was used for all the analyses.

### Results

**Fig. 1:**
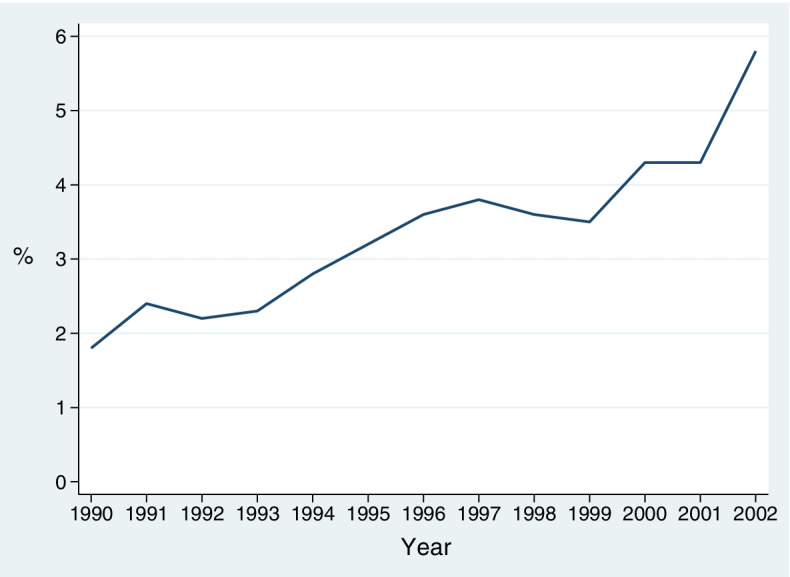
Incidence of OASI following vaginal delivery at Lillehammer Hospital, 1990–2002

During this 13-year period, 12,883 women gave birth at the study hospital, and 11,374 of them had a vaginal delivery. The mean incidence of first time OASI after vaginal delivery was 3.4% (*n* = 421), but the rate increased gradually from 1.9% in 1990 to a maximum of 5.8% in 2002 (Fig. 1). Of the 421 women with OASI and their matched controls, 275 (65%) were primiparous and 146 (35%) multiparous (104 had their 2^nd^, 33 their 3^rd^, 5 their 4^th^, 3 their 5^th^, and one her 6^th^ child). Both the primiparous and multiparous women with OASI differed similarly from their matched controls both on non-modifiable characteristics (larger size of the baby, higher GA, higher maternal age, and longer 1^st^ and 2d stage of labor) and modifiable characteristics (higher rates of amniotomy, augmentation of labor with oxytocin, and instrumental delivery). The multiparous women with OASI also had a higher rate of episiotomy (Table 1).

**Table 1:** Study characteristics of included primi- and multiparous cases and controls

	Primiparous women			Multiparous women		
	Case (*n* = 275) mean (SD) or n (%)	Control (*n* = 275) mean (SD) or n (%)	*P*	Case (*n* = 146) mean (SD) or n (%)	Control (*n* = 146) mean (SD) or n (%)	*P*
**Non-modifiable variables**						
Birthweight, gram	3678 (512)	3468 (561)	< 0.001	3975 (541)	3608 (673)	< 0.001
Birthweight quartiles	275 (50%)	275 (50%)	< 0.001	146 (50%)	146 (50%)	0.002
< 3300 g	65 (11.8%)	102 (18.5%)		12 (4.1%)	32 (11.0%)	
3300–3659 g	63 (11.5%)	78 (14.8%)		31 (10.6%)	38 (13.0%)	
3660–4039 g	82 (14.9%)	54 (9.8%)		41 (14.0%)	36 (12.3%)	
≥ 4040 g	65 (11.8%)	41 (7.5%)		62 (21.2%)	40 (13.7%)	
Length, cm ^□^	51.1 (2.1)	50.4 (2.5)	< 0.001	51.9 (2.5)	50.8 (3.1)	< 0.001
Head circumference, cm^□^	35.2 (1.5)	34.9 (1.6)	< 0.001	36.1 (1.3)	35.3 (1.6)	< 0.001
Gestational age, weeks ^□^	40.3 (1.7)	39.8 (2.1)	0.002	40.5 (1.4)	39.8 (2.3)	0.002
Gestational age categories^□^	274 (50.6%)	268 (49.4%)	0.004	145 (51.1%)	139 (48.9%)	0.603
Preterm (< 37 weeks)	6 (1.1%)	16 (1.1%)		4 (1.4%)	7 (2.5%)	
At term (37–42 weeks)	234 (43.2%)	233 (43%)		131 (46.3%)	122 (43.0%)	
Post term (> 42 weeks)	34 (6.3%)	19 (3.5%)		10 (3.5%)	10 (3.5%)	
Maternal age, years	26.7 (4.2)	25.7 (4.7)	0.004	31.0 (4.6)	29.6 (4.4)	0.005
Maternal age category	275 (50%)	275 (50%)	0.014	146 (50%)	146 (50%)	0.087
< 25 years	83 (15.1%)	115 (20.9%)		11 (3.8%)	18 (6.2%)	
25–29 years	122 (22.2%)	107 (19.5%)		46 (15.8%)	57 (19.5%)	
> 30 years	70 (12.7%)	53 (9.6%)		89 (30.5%)	71 (24.3%)	
Fetal presentation ^□^	275 (50%)	275 (50%)	0.116	146 (50.2%)	145 (49.8%)	0.365
Occiput anterior	257 (46.7%)	257 (46.7%)		134 (46.1%)	138 (47.4%)	
Deep transverse	0	1 (0.2%)		0	1 (0.3%)	
Occiput posterior	14 (2.6%)	7 (1.3%)		9 (3.1%)	4 (1.4%)	
Breech	4 (0.7%)	10 (1.8%)		3 (1.0%)	2 (0.7%)	
Duration of 1^st^ stage, minutes ^□^	368.5 (195.8)	314.8 (186.3)	< 0.001	268.4 (163.1)	197.3 (154.9)	< 0.001
Duration of 2^nd^ stage, minutes	46.6 (27.5)	41.4 (25.1)	0.037	29.3 (22.3)	19.2 (17.3)	< 0.001
**Modifiable variables**						
Induction of labor	26 (9.5%)	14 (5.1%)	0.049	13 (8.9%)	10 (6.9%)	0.515
Amniotomy	63 (22.9%)	18 (6.6%)	< 0.001	33 (22.6%)	11 (7.5%)	< 0.001
Augmentation with oxytocin	165 (60%)	119 (43.3%)	< 0.001	71 (46.7%)	36 (23.7%)	< 0.001
Episotomy^□^	190 (69.1%)	171 (62.2%)	0.155	66 (45.2%)	34 (23.3%)	< 0.001
Maternal birth position^□^	275 (51.0%)	264 (49.0%)	0.596	146 (51.2%)	139 (48.8%)	0.150
Supine/sitting	221 (41.0%)	203 (37.7%)		111 (39.0%)	97 (34.0%)	
Side bearing	19 (3.5%)	29 (5.4%)		12 (4.2%)	20 (7.0%)	
Standing	2 (0.4%)	2 (0.4%)		6 (2.1%)	4 (1.4%)	
Kneeling	15 (2.8%)	14 (2.6%)		5 (1.8%)	11 (3.9%)	
Stool	18 (3.3%)	16 (3.0%)		12 (4.2%)	7 (2.5%)	
Instrumental delivery						
Vacuum extraction	52 (18.9%)	22 (8.0%)	< 0.001	17 (11.6%)	3 (2.1%)	0.001
Forceps	41 (14.9%)	18 (6.6%)	0.002	8 (5.5%)	1 (0.7%)	0.018

Data are presented as mean (SD) or n. ^□^Missing data: Length = 15; Head circumference = 10; Gestational age = 16; Fetal presentation = 1; Duration of 1^st^ stage = 1; Episotomy = 2; Maternal birth position = 18

In the final multivariate conditional logistic regression model, higher maternal age and birthweight for the primiparous women and birthweight for the multiparous women were the only non-modifiable variables associated with rates of OASI (Tables 2 and 3).

**Table 2:** Multivariate conditional logistic regression analysis for obstetric anal sphincter injury among primiparous women

Variable	n	Crude		Adjusted	
**Non-modifiable variables**					
Birthweight quartiles	550				
< 3300 g	167	Reference		Reference	
3300–3659 g	141	1.22	0.77–1.96	0.90	0.52–1.55
3660–4039 g	136	2.36	1.47–3.81	2.27	1.31–3.93
≥ 4040 g	106	2.42	1.45–4.01	2.13	1.18–3.84
Maternal age category	550				
< 25 years	198	Reference		Reference	
25–30 years	229	1.57	1.07–2.29	1.68	1.08–2.62
> 30 years	123	1.87	1.17–2.99	2.04	1.17–3.57
**Modifiable variables**					
Amniotomy	81	4.00	2.27–7.04	4.84	2.60–9.02
Augmentation with oxytocin	284	1.98	1.39–2.81	1.63	1.08–2.46
Instrumental delivery					
Vacuum extraction	74	2.76	1.59–4.81	1.91	1.03–3.57
Forceps	59	2.64	1.42–4.89	2.37	1.14–4.92

R^2^ = 0.22

Data are presented as odds ratio with 95% confidence intervals

**Table 3:** Multivariate conditional logistic regression analysis for obstetric anal sphincter injury among multiparous women

Variable	n	Crude		Adjusted	
**Non-modifiable variables**					
Birthweight quartiles	292				
< 3300 g	44	Reference		Reference	
3300–3659 g	71	3.42	1.24–9.46	5.58	1.56–20.0
3660–4039 g	80	4.91	1.70–14.2	6.90	1.86–25.7
≥ 4040 g	109	7.13	2.57–19.8	11.4	3.10–42.2
**Modifiable variables**					
Amniotomy	44	3.44	1.64–7.23	3.76	1.45–9.76
Augmentation with oxytocin	107	3.19	1.82–5.59	3.70	1.79–7.67
Episiotomy^a^	101	2.88	1.66–5.00	2.64	1.36–5.14

R^2^ = 0.28

Data are presented as odds ratio with 95% confidence intervals

^a^n is reduced by 2 from 292 to 290 due to missing data on episiotomy

Of the modifiable variables, amniotomy was strongly associated with OASI, both in the primiparous (OR 4.84, 95% CI 2.60–9.02) and multiparous (OR 3.76, 95% CI 1.45–9.76) women, as was augmentation with oxytocin (OR 1.63, 95% CI 1.08–2.46 and 3.70, 95% CI 1.79–7.67, respectively, Tables 2 and 3). Instrumental delivery was associated with OASI in the primiparous women (Table 2) and episiotomy with OASI in the multiparous women (Table 3). The same trend, but not statistically significant, was found for the non-modifiable and modifiable variables when limiting the conditional regression analysis to the subgroup of women who gave birth to their 3^rd^ or later child (*n* = 84). We found no significant interactions or multicollinearities.

## Discussion

In this unselected population, OASI was associated with known non-modifiable factors like high maternal age, first pregnancy, and large babies. Of potentially modifiable factors, OASI was associated with induction of labor and instrumental vaginal delivery in primiparous women, and with amniotomy and augmentation with oxytocin in both primi- and multiparous women, procedures that are primarily initiated to accelerate delivery.

The major strengths of this study were the unselected population, the large number of participants, and completeness of data. We also consider the inclusion of only one obstetric hospital a strength since no major official changes in routines were introduced, although we cannot exclude gradual unrecognized changes during this 13-year period. The retrospective nature of the study may be a weakness since reasons for performing amniotomy and augmentation with oxytocin were not necessarily specified and since vigilance in classifying perineal rupture may have been less accurate than in a planned prospective study. Furthermore, we have no data on OASI diagnosed after discharge from the hospital. The time lap between the collection and publication of data may make the results less valid of today’s practice since increased focus on reducing the incidence of OASI has been implemented since the data were collected [13, 16]. According to the Medical Birth Registry of Norway, there has been a reduction in the proportion of women experiencing OASI after vaginal birth since our study was conducted (1.6 in 2020 vs 4.6 in 2003) [25]. This decrease has occurred even though the proportions of labor inductions and augmentation with oxytocin have increased nationally in the same period [25]. We speculate that a more cautious use of amniotomy may have contributed to the decline since our results became widely known in Norway at the time when the data were collected.

To our knowledge, our study is the first to include amniotomy as a potential independent risk factor for OASI. A recent meta-analysis on risk factors for severe perineal trauma in child birth only identified one study that addressed the potential role of amniotomy [1]. However, the significance of amniotomy per se could not be assessed because it was combined with the use of oxytocin for augmentation of labor. In our study, amniotomy was the strongest independent modifiable risk factor regardless of parity and suggests that attention to indications and timing of amniotomy may be a hitherto unrecognized means of preventing OASI. The use of amniotomy varies between institutions both in Norway and other countries and ranges from 20 to 60% [26, 27]. However, in our experience the documentation of amniotomy in patient charts during labor is highly variable. Even though we have a national high-quality birth registry in Norway, the use of amniotomy in spontaneous labor has not reported since 1998 [25].

With the goal of reducing cesarean births through active management of labor, amniotomy has been widely and readily accepted to avoid labor for more than 12 h [28]. However, reducing length of labor might not be a benefit for all women, and a Cochrane review from 2013 concluded that there is no evidence to support routine amniotomy to shorten spontaneous labor or to avoid prolonged labor [29]. The mechanism behind the association between amniotomy and OASI is unclear, but we speculate that amniotomy may disrupt the normal physiologic process of gradual adaptation of the birth canal and thereby a higher risk of trauma. Thus, our findings indicate that untimely use of amniotomy may act in line with other established indicators of pathologic birth mechanics such as high birth weight, large head circumference, fetal occiput presentation, prolonged second stage of labor, augmentation with oxytocin, episiotomy, and instrumental delivery [1].

In the present study, we also found that augmentation with oxytocin was an independent risk factor for OASI for both primi- and multiparous women. This is in accordance with previous studies [1]. Augmentation with oxytocin is widely used when labor is delayed, and probably more than half of women in labor worldwide receive oxytocin augmentation [25, 27]. However, the use of augmentation varies widely between countries and within the same country. In our study, 60% of the primiparous and 47% of the multiparous women were augmented with oxytocin, which is in line with current rates in maternity wards in Norway [27]. Increased frequency and intensity of contractions are known potential adverse effect of augmentation of labor with oxytocin [30]. We suggest that the effects of augmentation with oxytocin are similar to that of amniotomy in that the birth progress may be more rapid than the natural adaptation of the birth canal.

Instrumental vaginal delivery is a well-established risk factor for OASI [1, 2, 24]. However, this was only an independent risk factor in primiparous women in our study. Instrumental delivery was also associated with OASI in multiparous women in the unadjusted analysis, and the reason for no significant association in the adjusted analysis may partly be that the study lacked power to detect a risk since instruments were rarely used in this group.

In conclusion, the study suggests that indications for and timing of amniotomy and augmentation of the birth process with oxytocin need to be readdressed in order to reduce the risk of severe perineal ruptures.

## Data Availability

The dataset generated and analysed during the current study are not publicly available due to restrictions in data-sharing of the institutional perinatal database but are available from the corresponding author on reasonable request, MNH-A, upon reasonable request.

## Abbreviations

CI: Confidence interval
OR: Odds ratio
OASI: Obstetric anal sphincter
